# The Effect of Active Additives and Coarse Aggregate Granulometric Composition on the Properties and Durability of Pervious Concrete

**DOI:** 10.3390/ma15031035

**Published:** 2022-01-28

**Authors:** Vilma Banevičienė, Jurgita Malaiškienė, Renata Boris, Jiri Zach

**Affiliations:** 1 Laboratory of Composite Materials, Faculty of Civil Engineering, Institute of Building Materials, Vilnius Gediminas Technical University, 10223 Vilnius, Lithuania; vilma.baneviciene@vilniustech.lt (V.B.); renata.boris@vilniustech.lt (R.B.); 2 Institute of Building Materials and Components, Faculty of Civil Engineering, Brno University of Technology, 60200 Brno, Czech Republic; zach.j@fce.vutbr.cz

**Keywords:** pervious concrete, active additives, coarse aggregate size, portlandite, physical mechanical properties, infiltration rate, sound absorption

## Abstract

Pervious concrete (PCO) has many advantages and applications, such as water pooling reduction, noise attenuation, replenishment of groundwater reserves, etc. However, the use of pervious concrete is limited due to its low compressive strength and durability, especially as a result of portlandite leaching from concrete exposed to flowing water. The effects of active additives (nano SiO_2_ (NS) spent catalyst generated at the fluid catalytic cracking unit (FCCCw) and paper sludge waste burned at 700 °C (PSw)) along with particle size distribution of the coarse aggregate on the properties and durability of pervious concrete were determined in the research. Active additives used in the binder were found to reduce portlandite leaching from concrete exposed to flowing water to significantly increase the resistance of concrete to freezing and thawing cycles and to increase sound absorption, compressive strength and infiltration rate. In addition, industrial waste (FCCCw and PSw) used as active additives significantly reduced the use of clinker in concrete applied in the construction of water pervious systems. The coarse aggregate size distribution had the greatest effect on the density, ultrasound pulse velocity (UPV), porosity, compressive strength and infiltration rate of pervious concrete.

## 1. Introduction

Pervious concrete (PCO) is most often used in locations with limited traffic and load: pedestrian areas, bicycle routes, car parks, zoo paving, bridge embankments, solar energy storage systems, tide water control, etc. [1,2]. The advantage of pervious concrete is the ability to control temperature and humidity of pavements [3,4]. Pervious concrete is lighter and usually contains a lower amount of binder (cement); therefore, less energy is used to produce such concrete [5,6]. PCO has sound absorbing properties and can be used for the construction of noise barriers or as a paving for storm water treatment [7,8,9,10,11,12,13,14]. Airborne sound insulation and sound absorption in the soundproofing band of frequencies from 100 to 5000 Hz are the key parameters for the design and construction of noise barriers [15]. Irrespective of numerous advantages of PCO, such as noise abatement, recharging groundwater, reducing urban heat island effect, and the application of such concrete is limited due to its low compressive strength, low durability and insufficient regulation [16].

PCO is coarse-grained concrete usually consisting of coarse aggregates, binder, water and chemical admixtures [17]. Fine aggregates are either not used or used in small amounts in pervious concrete mixes [18,19]. The standard aggregate fraction is 10/20 for ordinary PCO in order to maintain a sufficient void content [20,21,22]; however, aggregates of fraction 5/10 are also used to improve mechanical properties of pervious concrete [23,24]. A large number of voids makes pervious concrete relatively lightweight (density ranges between 1600 and 1900 kg/m^3^). According to [25], the porosity of PCO ranges from 15% to 35%, and the compressive strength ranges from 2.8 to 28 MPa [17,26,27]. The flow rates of water flowing through pervious concrete differ depending on the aggregate particle size and density of the mix, but usually it ranges between 504 and 4392 cm/h. Other authors state that the average open porosity of hardened pervious concrete is between 15% and 25%, whereas the rate of water flow is between 488 and 4379 cm/h [27]. The authors of [28] found that the flowability of PCO with an aggregate particle size of 9.5 mm gave better results compared to PCO with aggregate particle size of 19 mm. The authors of [29,30] also determined that smaller-size aggregates increase the compressive strength and reduce water permeability. Bigger-size aggregates increase water permeability and deteriorate sound absorption properties [2,31]. The paper in [32] describes a study of eight compositions of pervious concrete with similar porosity but different aggregate sizes. Special image analysis technology was used to investigate the 2D/3D structure of permeable concrete voids and to test the effect of porosity on water permeability. The results of the study revealed that PCO specimens with almost the same porosity had different water permeability values. The water permeability values were found to increase with a bigger particle size of the coarse aggregate. It was also noted that water permeability intensifies with higher porosity and slows down when the volume of voids reaches 500 mm^3^. A positive correlation was found between water permeability and the ratio of the total pore volume and specific surface area. Passing through the pores with a higher ratio of total pore volume to specific surface area, the water experiences less friction against the pore walls, and permeability is improved. Irrespective of the aggregate particle size, there will always be a significant number of small pores (< 2 mm^2^) in pervious concrete. The correlation of small pore size with small pore volume will be negative. Pervious concrete mixture is stiff; slump is 10–50 mm [27]. PCO strength can be increased by strengthening the cement-based matrix, i.e., by using pozzolanic additives combined with micro-fillers. However, the increased strength reduces water permeability of concrete. Researchers [33,34,35] have found that a higher compressive strength of pervious concrete is obtained by using coarse aggregates of different fractions, rather than by a single fraction, because the strength of pervious concrete is dependent on the size of the pores and the pore volume, which is reduced by aggregates of different particle sizes [36]. Various nano-components can be used to increase the compressive strength and durability of concrete, such as nano SiO_2_ [37,38,39,40], fly ash [41], pelletized blast furnace slag [42], various types of polymeric fibre [31,34,41], and microfillers [43]. Nano SiO_2_ has a big particle surface area and high pozzolanic activity, and thus speeds up the reaction with calcium hydroxide, increases the resulting amount of calcium hydrosilicates (CSH) [44,45], and increases early compressive strength [46,47,48]. However, due to the differences in the structure of ordinary and pervious concrete, the increase in strength is not significant in PCO [33,43].

Some results reported in research articles on pervious concrete are shown in Table 1.

The results in Table 1 show that researchers use a variety of waste materials with pozzolanic properties (fly ash, blast furnace slag) and inert properties (recycled concrete and asphalt) for pervious concrete. The porosity and compressive strength of such concrete, as described above, varied from approx. 16% to 32% and from approx. 3 to 26 MPa, respectively. The water permeability varied over a wide range from 432 to 9468 cm/h. Pervious concrete with such properties can be used for light traffic pavement, but it is not suitable for heavy-duty pavement solutions [54].

The binder content in concrete mix is also important for the compressive strength and structure of pervious concrete. An excessive amount of the binder can fill voids and reduce porosity, while an insufficient amount of the binder will reduce the binder coating around the aggregate particles and reduce the compressive strength of the mix. The optimum content of cement-based materials depends on aggregate particle size and distribution [22,55]. Tests [56] showed that W/C of 0.26–0.45 ensures the adequate covering of the aggregate with the binder and ensures binder stability. The aggregate-to-binder ratio in pervious concrete ranges between 4:1 and 6:1 [5]. The strength increases with the lower aggregate-to-binder ratio and vice versa [38,57]. Three batches of concrete specimens made of different size aggregates when the aggregate-to-cement ratio is 6:1, 8:1, and 10:1, and when no fine aggregate is used are analysed in the paper [3]. Concrete mixes with a higher aggregate to cement ratio of 8:1 and 10:1 were found to be suitable for pavement that does not require high compressive strength but requires high water permeability. The compressive strengths of concrete with the same aggregate-to-cement ratio (6:1, 8:1, and 10:1) as follows, 39%, 29%, and 26% [58], was calculated according to the control sample. Another paper [21] reports the maximum value of 28 MPa achieved with the ratio 3:8. The results of the tests in [39,42,58], with a low aggregate-to-binder ratio (2.50, 2.48, 2.58), showed that the strength did not always increase with reduced porosity of concrete. Concrete made with a flowable binder may lose performance characteristics because the binder will concentrate on the bottom of the specimen [59]. The recommended W/C in [34] is from 0.27 to 0.34. The authors of [60] studied the effect of W/C ratio (0.30, 0.35, 0.38) and aggregate size on the properties of pervious concrete. They found that the mixtures made of 30% 10 mm size and 70% 4.75 size aggregates produced pervious concrete suitable for paving with a porosity between 24% and 26%, density between 1923 and 1985 kg/m^3^, and compressive strength between 13.4 and 17.5 MPa at 28 days. With the increase in W/C ratio, the compressive strength decreased, and the binder settled at the bottom of the specimens, thus preventing the flow of water. The authors of [61] determined that lower W/C increases the compressive strength of concrete, but a low amount of water can cause deterioration in the concrete surface.

The compaction technique and parameters (pressing under controlled compaction force, vibro-compaction by controlling frequency and time) is another important factor that has had an effect on the properties of pervious concrete. According to [62], the aim of compaction is to strengthen the connection between the aggregates and the binder. However, a too intensive compaction may ruin the structure of voids and reduce the porosity and thus the permeability of concrete [29].

Durability of concrete is one of the most important issues in the design of new structures and assessment of the state of existing structures [63,64,65]. As mentioned above, the size of the aggregate, the aggregate-to-binder ratio, the water–cement ratio and the method of compaction are the factors that have the greatest effect on the design properties and durability of water-pervious concrete pavements.

The aim of this research work is to analyse the complex effect of active additives (nano SiO_2_, spent catalyst generated at the fluid catalytic cracking unit (FCCCw), and paper sludge waste burned at 700 °C (PSw)) and the particle size distribution of the coarse aggregate on the properties and durability of pervious concrete.

## 2. Materials and Methods

The following materials were used for the tests: cement CEM I 42.5 R manufactured by Heidelberg Cement (Rocket cement M-600, Heidelberg Cement, Skovde, Sweden); spent catalyst generated at the fluid catalytic cracking unit of oil refinery (FCCCw) (AB Orlen Lietuva, Mazeikiai, Lithuania); nano SiO_2_ (NS) in the form of white powder (Sigma-Aldrich, Taufkirchen, Germany); paper industry waste generated at AB Grigeo, Lithuania (PSw).

Chemical compositions of cement, FCCCw, and PSw fired at 700 ^o^C are presented in Table 2. Mineral composition of the cement used: 56.6% of C_3_S, 16.7% of C_2_S, 9.0% of C_3_A, 10.6% of C_4_AF, and 7.1% of other materials. Physical and mechanical properties of the cement used: density 3.1 g/cm^3^, initial setting time 180 min., compressive strength at 28 days 55 MPa.

**Figure 1:**
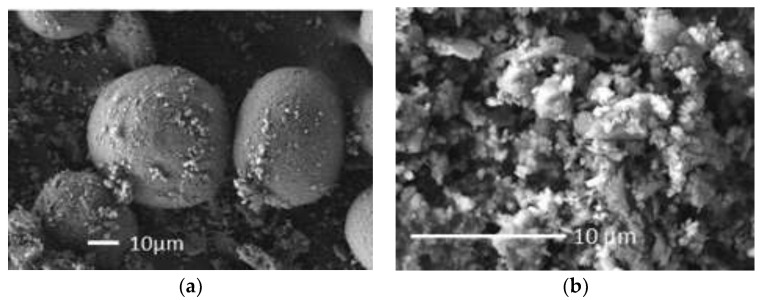
Images of FCCCw (**a**) and PSw particles (**b**).

Figure 1a illustrates the scanning electron microscopy (SEM) image of FCCCw particles (density 2.4 g/cm^3^). FCCCw particles are almost spherical with a rough surface. Figure 1b illustrates the SEM image of PSw particles (density 2.5 g/cm^3^), which vary in size, have irregular form and tend to agglomerate.

**Figure 2:**
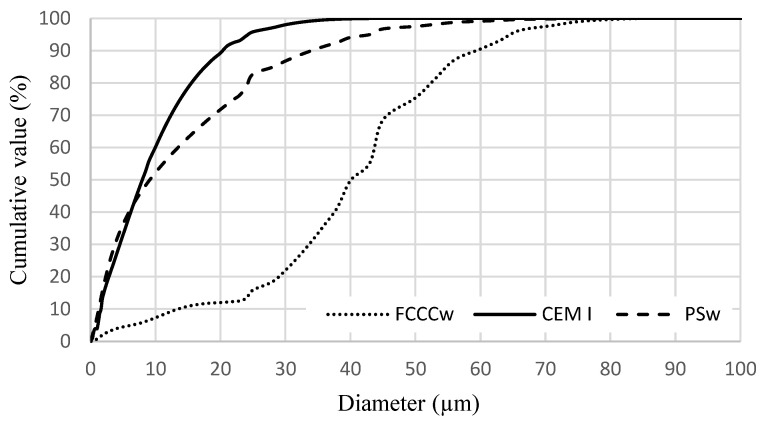
Cement, FCCCw and PSw particle size distribution.

Figure 2 illustrates the distribution of cement, FCCCw, and PSw particle sizes. Cement, FCCCw and PSw particle size d_50_ is 10.3, 43.1, and 9.1 µm, respectively; particle size d_90_ is 22.9, 62.5, and 36.0 µm, respectively. The average diameter of cement, FCCCw and PSw particles is 11.7, 40.0, and 14.5 µm, respectively.

**Figure 3:**
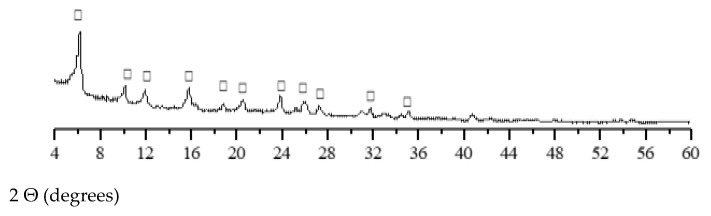
XRD pattern of FCCCw (□—faujasite).

**Figure 4:**
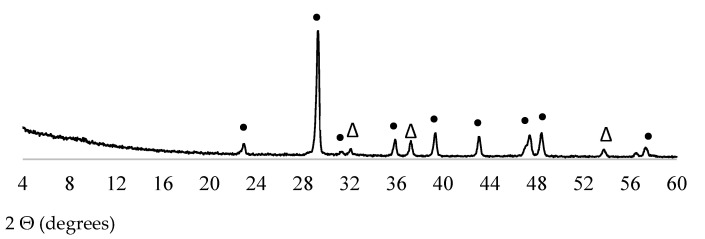
XRD pattern of PSw (•—calcite, ∆—CaO).

X-ray diffraction (XRD) patterns of FCCCw and PSw are presented in Figure 3 and Figure 4, respectively. Y-type zeolite faujasite is the main crystal phase in FCCCw [66]. Calcite and CaO were the two main minerals identified in PSw.

FCCCw was selected for the composition of pervious concrete for its pozzolanic properties [67]. PSw was selected for its micro-filler function and reactivity that changes cement hydration products and reduces the porosity of cement-based materials and water permeability of the binder [68,69].

The properties of NS were as follows: size 10–30 nm; surface area 202 m^2^/g; SiO_2_ purity 99.8%; pH (20% solution) 4.0; density 2.2 g/cm^3^. Physical–mechanical properties of the coarse aggregate (gravel) are presented in Table 3. Chemical composition of the gravel (by fractions) is presented in Table 4.

Melamine-based superplasticiser (SP) in powder form, pH 9.4 (20% solution) was used. Tap water was used to prepare the specimens. Seven pervious concrete mixes (Table 5) were made from the materials listed above (Table 2, Table 3 and Table 4), water–binder ratio (W/B) was 0.35, and aggregate–binder ratio (A/B) was 5.6:1. The coarse aggregate with the same particle size distribution was used in PCO-C and PCO-1, but the composition of the binder was different. PCO-C composition contained only cement, and other compositions were mixed with a composite binder modified with active additives (NS, FCCCw, and PSw), which were developed in previous research works [66,67]. This binder described in previous papers was developed with the aim to use it in the manufacturing of pervious concrete for the following reasons: the complex application of 10% FCCCw, 2.5% PSw and 0.02% NS produced the best compressive strength results; the lowest level of portlandite was observed (portlandite is the most susceptible to dissolution in the presence of water); the highest levels of CSH and CASH were identified; the highest rate of hydration was observed. The amount of the superplasticizer was calculated according to the amount of the binder, which was constant at 270 kg/m^3^. The binder content in pervious concrete is quite small, as it is only needed for the adherence of the coarse aggregate. The first two compositions (PCO-C and PCO-1) were used to find out how the application of a combined binder change the properties and durability of pervious concrete. Other compositions were used to evaluate the effect of particle size distribution of the coarse aggregate on the properties of pervious concrete with active additives. The slump of all mixtures was less than 1 cm.

Two types of specimens were formed from the mixture compositions given in Table 5. Specimens of size 150 × 150 × 150 mm^3^ were used to measure sound absorption properties and 100 × 100 × 100 mm^3^ sizes specimens were used to measure other properties analysed in this paper.

At first, the binder was mixed with water in a Hobart-type mixer. The binder containing only cement was mixed according to the standard procedure (EN 196-1). When FCCCw and PSw are added to the binder, the additions are first mixed with cement for 90 s and then the mixture is mixed with water according to the standard procedure (EN 196-1). NS was homogenised with one part of water using an ultrasonic disperser and then mixed with the remaining water. The coarse aggregate was added to the binder mixed with water and mixed for 180 s. The mixture was placed into moulds and pressed by placing a 15 kg weight on top of each moulding form. The specimens were cured in metal moulds at 20 °C in 95% humidity for two days. Afterwards, the demoulded specimens were cured in water of 20 °C for 26 days.

The density of the specimens in solid state (3 specimens from each batch) was calculated from the specimen’s weight (0.01 g precision) and volume calculated from the specimen’s dimensions (0.01 mm precision). Measurements were taken after 7 and 28 days of curing. Ultrasonic pulse velocity (UPV, m/s) was calculated according to the literature [67].

The compressive strength of pervious concrete was determined according to EN 12390-3:2003.

Water infiltration rate was determined according to reference [70]. The obtained result was converted into cm/h.

The porosity of pervious concrete was calculated according to two methods: (1) assessment of the density of the material in solid state and in natural state (total porosity, %); (2) calculation of pores in the cross-section of the specimen by means of Cool PHP Tools software.

With the aim to determine the effect of active additives on the dissolution of portlandite, cut and polished pieces of the specimens were tested by exposing them to cyclic water flow of 8 h for 15 days, 120 h in total (10 l of circulating water were used). XRD analysis of the specimens and SEM and EDS analyses of the cut and polished pieces were performed before and after the test. Water was also analysed for calcium content, pH and electric conductivity before and after the test. pH Electrode (InLab 410) with the accuracy of 0.01 was used in combination with the conductivity probe with the range of 0.01‒1000 µS/cm (InLab 730) for pH and electrical conductivity measurements, respectively. The measurements were taken at 20 ± 1 °C temperature.

The microstructure of the samples was analysed on the scanning electron microscope (SEM) JSM-7600F (JEOL, Tokyo, Japan). The analysis was performed at accelerating voltage 4 or 10 kV, the mode of secondary electrons was used in image formation. Before the investigation, the surface to be investigated was covered with a layer of electrical conductor using a sputtering device QUORUM Q150R ES (Quorum Technologies, Laughton, UK). X-ray microanalysis was performed by the energy dispersion spectrometer (EDS) Inca Energy 350 (Oxford Instruments, Abingdon, UK), using Silicon Drift type detector X-Max20. The INCA software package (Oxford Instruments) was used.

X-ray diffraction (XRD) analysis of the phase composition of materials was performed according to the method described in literature [67].

Freeze–thaw resistance was tested according to LST 1428-17 by measuring the diminishing strength and loss of mass after freezing and thawing cycles. The compressive strength may not decrease more than 5%, and the loss of mass may not exceed 3%.

The single-digit value of the sound absorption factor was determined according to EN ISO 10534-1 in the third octave bands. On the basis of these values measured according to ISO 10534-1, a single value of sound absorption was determined according to EN 1793 [15]. The sound absorption coefficient was calculated from the pressure variation of the standing sound wave (maximum and minimum values) [71].

## 3. Results and Discussion

**Figure 5:**
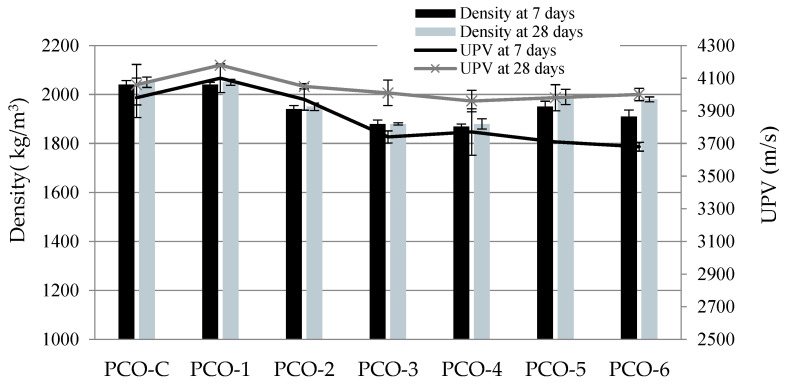
Density and UPV results of pervious concrete.

**Figure 6:**
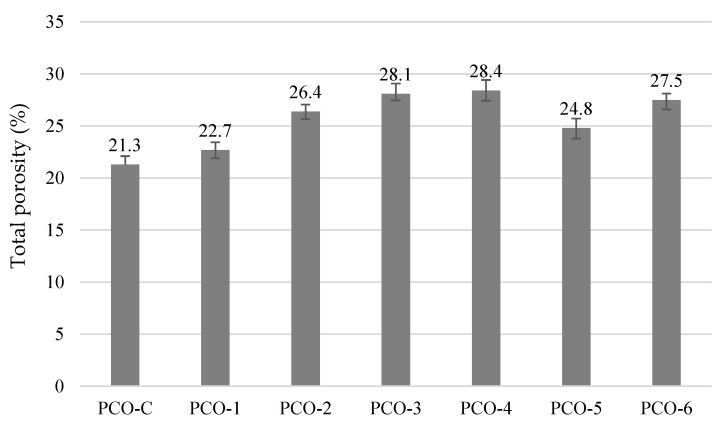
Total porosity of pervious concrete.

The highest density (2050 kg/m^3^) and UPV (~4180 m/s) values of pervious concrete at 28 days were obtained when equal parts of all gravel fractions were used (Figure 5), as such a distribution of particle sizes resulted in the lowest porosity of concrete (Figure 6). Researchers had also found [32] that density and porosity reduce when different size aggregates are used because finer aggregates fill the spaces between coarse aggregate particles. Almost no difference was observed between the density and UPV values of reference specimens (PCO-C) and PCO-1 specimens of the same particle size distribution and with active additives because density and UPV mainly depend on the particle size distribution of the coarse aggregate. The lowest density value (1880 kg/m^3^) was obtained in compositions PCO-3 and PCO-4, where the coarse aggregate of either 4/8 or 8/16 was used. Density difference at 7 and 28 days was not significant because a low amount of the binder was used, and the difference was caused by the distribution of the coarse aggregate in the specimen.

The highest total porosity value (Figure 6) was obtained for PCO-3 and PCO-4 specimens, where aggregates of only one fraction were used. These compositions also had the highest porosity of approx. 16% in the cross-section of the specimens (Table 6). The less porosity in cross-section can be explained: (1) porosity of binder matrix was not evaluated; (2) uneven distribution of pores throughout the sample.

The total porosity of PCO-3 and PCO-4 increased 24% and approx. 40% in the cross-section, compared to PCO-C. The biggest number of coarse voids (8.8%) was observed in composition PCO-4 where the aggregates of the biggest fraction 8/16 were used, and the biggest voids formed among the coarsest particles. Active additives, although used in a small amount, also influenced the porosity. The lowest porosity values were obtained in specimens PCO-C where pure cement was used. The comparison of PCO-C and PCO-1 porosity images revealed a thicker coating of the aggregates by a binder film.

**Figure 7:**
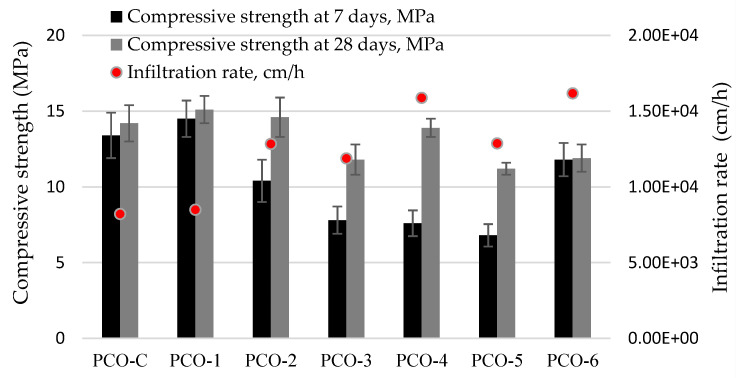
Results of compressive strength and infiltration rate of pervious concrete.

The results of water permeability measurement (Figure 7) showed that water permeability is higher (12860–16180 cm/h) and compressive strength is lower (11.2–13.9 MPa) in specimens with bigger voids and a higher number of voids (PCO-3, PCO-4, and PCO-6). The highest compressive strength (15.1 MPa) was found in the specimens where gravel of all fractions was added equally; however, water permeability of those specimens was the lowest, although it was still quite high (8500 cm/h) and sufficient. The active additives used had a positive effect on the compressive strength and water permeability values. With the reduced cement content and active additives added, the compressive strength of pervious concrete increased 6.3% and water permeability increased 3.5%. The effect of these additives on the properties and hydration of hardened cement paste was reported in research publications [66,67]. According to the published results, NS, FCCCw and PSw used together accelerate cement hydration by 4 h, increase the compressive strength by approx. 40%, reduce the size of the binder pores by approx. 20%, reduce the amount of portlandite by 30%, and increase the amount of CSH. The compressive strength of PCO increased because of increased CSH content and decreased number of voids in the binder.

**Figure 8:**
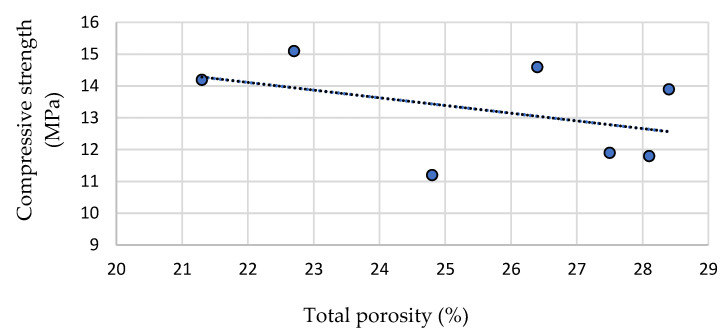
The effect of total porosity on compressive strength.

Figure 8 illustrates the relationship between compressive strength and total porosity. The trendline is standard and confirms that compressive strength decreases with the increase in total porosity. However, the dissipation of dots shows, that in this case, the compressive strength was related not only with the total porosity but also with the framework formed by the coarse aggregate, the strength of the aggregate and the effect of active additives compared to PCO-C and PCO-1.

The results of physical and mechanical properties of water-permeable concrete show that the size of coarse aggregate particles has the greatest effect on density, UPV, porosity, water permeability and compressive strength, leading to the formation of the porous concrete structure. Active additives have a slight positive effect on the strength and water permeability of concrete. However, further tests showed that active additives play an important role in the durability analysis of pervious concrete.

**Figure 9:**
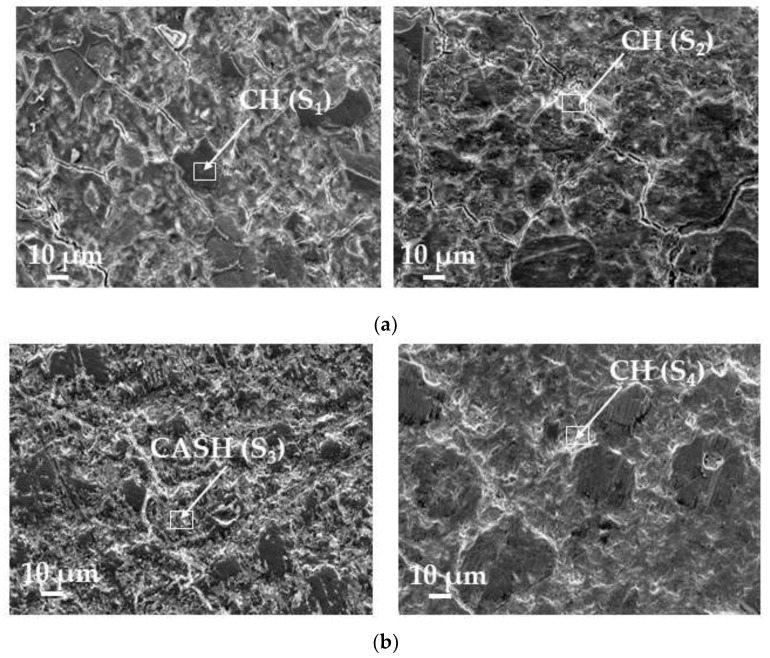
SEM images of cut and polished specimens before (**a**) and after (**b**) the exposure to the stream of water. (**a**) PCO-C→PCO-1. (**b**) PCO-C→PCO-1.

**Figure 10:**
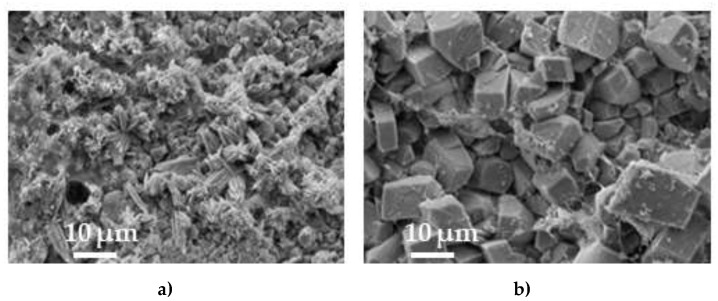
Images of cut and polished pieces after the exposure to a water stream without washing the surface coat: (**a**) PCO-C; (**b**) PCO-1.

Portlandite dissolution under a long-term exposure to a stream of water is one of the problematic issues in pervious concrete applications. SEM and EDS analyses were performed to evaluate the dissolution of portlandite before and after the exposure of the specimen to water flow (Figure 9 and Figure 10; Table 7 and Table 8).

EDS analysis of areas S_1_ and S_3_ in PCO-C specimens showed that after the exposure to water flow, Ca content decreased 37%, the colour of the area S_3_ had changed compared to the area S_1_ prior to the washing, and the layer of cementitious matrix in this area reduced. These results suggest that some portlandite leached out and CASH from a deeper layer was seen in area S_3_. EDS analysis of areas S_2_ and S_4_ in PCO-1 specimens showed that Ca content decreased 17% and the appearance of the area remained the same. These results suggest that less portlandite leached out compared to specimen PCO-C. The active additives used not only reduced the amount of portlandite in the cement matrix, as found in reference sources, but also created a protective layer of calcite, which buffered the negative effect of water flow.

The results of the XRD analysis (intensities of the main peaks) presented in Table 8 show the change in the mineral composition of the reference specimen PCO-C of pervious concrete after the exposure to the water stream: the intensities of portlandite, alite and belite are lower. After the exposure to the water stream, the intensities of the same minerals in the specimen with active additives remained similar to those before the exposure. Water parameters measured before and after the test of the reference specimen (Table 9) showed that, after the test, water pH increased 7.1%, electric conductivity increased 2.2 times, and CaO content in the water increased 11 times. After the test of the specimen with active additives, water pH increased 6%, electric conductivity increased 1.6 times, and CaO content in the water increased 8 times. These results suggest that active additives (NS, FCCCw and PSw) reduced the dissolution of portlandite and other minerals. The explanation is that active additives accelerated the formation of CSH, which is more resistant to dissolution, and made the structure of the cement matrix denser [66,67].

The results of freeze–thaw resistance tests of the selected characteristic compositions after 50 freeze–thaw cycles in water are given in Table 10. The data in the table show that active additions had the most significant effect on the freeze–thaw resistance, as they significantly increased the durability of concrete subjected to freezing and thawing cycles. The compressive strength of the specimens with active additives decreased 4.7% and actually withstood 50 cycles, while the compressive strength of reference specimens made of cement only decreased 31% (6.6 times difference) compared to the limit value of 5% prescribed by the standard (LST 1428-17).

**Figure 11:**
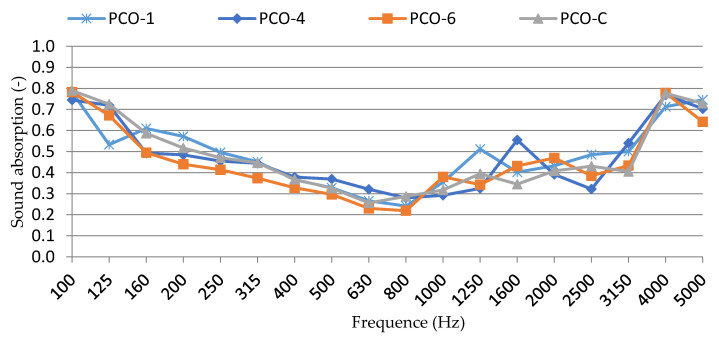
Overview of measured sound absorption values at given frequencies.

Sound absorption values at given frequencies for pervious concrete of selected compositions are presented in Figure 11 and Table 11.

There was no apparent relationship between the detected values, only that the sound absorption value for the composition PCO-1 was slightly higher than 2.3 dB. The other values were around 2.1 dB (Table 11). The comparison of results obtained for specimens PCO-C and PCO-1 showed that active additives increased the sound absorption by approx. 10%. The results suggest that, at a similar coarse aggregate particle size distribution, the binder with active additives has a thinner layer and sound propagation is inhibited in the pores.

## 4. Conclusions

A combined binder incorporating NS, FCCCw and PSw used in pervious concrete increased the compressive strength by 6%, while the density and UPV remained the same. The particle size distribution of the aggregate used in the mix of pervious concrete, which also significantly changed the porosity of concrete, had the greatest effect on the physical and mechanical properties of pervious concrete. The highest porosity of pervious concrete (28.4%) was obtained when the coarse aggregate of fraction 8/16 only was used. The mix also produced the highest water permeability of 16000 cm/h, but lower compressive strength of 12 MPa. The use of three fractions (2/4; 4/8, 8/16) of the aggregate in equal parts increased the compressive strength to 15 MPa and reduced the water permeability to 8500 cm/h with a porosity of 22.7%.

After exposure to the flow of water, lower intensities of portlandite, alite and belite were identified in the mineral composition of pervious concrete specimen PCO-C, where only cement was used as a binder, whereas the intensities in the specimen with active additives (NS, FCCCw, PSw) remained similar. Water pH after cyclic washing of PCO-C increased by 7.7%, the electric conductivity increased by a factor of 2.2 and the CaO content of the water increased by a factor of 11. In PCO-1 specimens with active additives, the pH of the circulating water increased by 6.4%, the electric conductivity increased by a factor of 1.6, and the CaO content increased by a factor of 8.

The assessment of the durability of pervious concrete in terms of freeze–thaw resistance showed that, irrespective of the coarse aggregate particle size distribution, the compressive strength of the specimens with active additives reduced by 4.7%, and actually withstood 50 freeze–thaw cycles. Meanwhile, the compressive strength of PCO-C specimens reduced by 31% after 50 freeze–thaw cycles (6.6 times more compared to the specimen with active additives).

The comparison of the results obtained for PCO-C and PCO-1 specimens showed that active additives increased the sound absorption by approx. 10%. The results suggest that, at a similar coarse aggregate particle size distribution, the binder with active additives has a thinner layer and sound propagation is inhibited in the pores.

Given that a major part of active additives used come from industrial waste (FCCCw) that does not require any additional treatment and that PSw is recycled industrial waste, the use of such materials contributes to the development of circular economy and the reduction of landfill waste. The cement content was reduced by 12.5%, and only 0.02% of SiO_2_ was used to keep the cost of permeable concrete low. The low amount of nanosilica was sufficient to obtain the desired effect.

## Figures and Tables

**Table 1:** The main properties of pervious concrete presented in the literature.

Used Additives	Porosity (%)	Compressive Strength After 28 Days (MPa)	Permeability (cm/h)	References
32% Fly ash	15.8	13.8	756	[49]
20% Fly ash	~32	7–9	432	[50]
50% Fly ash	~31	3–6	576	[50]
60% Blast furnace slag	26	13.0	–	[51]
Recycled concrete fillers (10–100%), 10% nano SiO_2_ and fibres	16.4–30.1	10.5–26	468–9468	[52]
10–100% of natural aggregate was replaced by recycled asphalt	23–25	3.7–12.8	1080–4320	[53]

**Table 2:** Chemical composition of cement, FCCCw and PSw (wt.%).

	CaO	SiO_2_	Al_2_O_3_	Fe_2_O_3_	MgO	K_2_O	Na_2_O	SO_3_	Cl	Others
Cement	63.2	20.4	4.0	3.6	2.4	0.9	0.2	3.1	0.05	2.2
FCCCw	0.5	50.1	39.4	1.3	0.5	0.1	0.2	2.3	0.01	5.6
PSw	88.0	5.1	3.8	0.6	1.2	–	0.2	0.4	0.06	0.6

**Table 3:** Properties of the coarse aggregate.

Property	Gravel Fraction		
	2/4	4/8	8/16
Bulk density (kg/m^3^)	1543	1476	1438
Particle density (kg/m^3^)	2670	2665	2670
Water absorption rate (%)	1.41	1.43	1.39

**Table 4:** Chemical composition of the gravel.

Gravel Fraction	Chemical Composition (%)								
	SiO_2_	CaO	Al_2_O_3_	MgO	Fe_2_O_3_	K_2_O	Na_2_O	TiO_2_	Other
2/4	40.4	38.8	7.8	6.0	2.1	2.7	1.2	0.2	0.8
4/8	25.9	50.9	6.1	9.5	2.4	2.4	0.9	0.2	1.7
8/16	26.6	55.9	5.6	6.3	2.2	1.8	0.8	0.2	0.6

**Table 5:** Compositions of pervious concrete for 1 m^3^ and slump test results.

Designation	Cement (kg)	Gravel (kg)			FCCCw (kg)	PSw (kg)	NS (g)	SP (kg)	Slump (cm)
		2/4	4/8	8/16					
PCO-C	270.0	500	500	500	0	0	0	0.5	0.8
PCO-1	236.2	500	500	500	27	6.75	54	0.5	0.8
PCO-2	236.2	0	750	750	27	6.75	54	0.5	0.6
PCO-3	236.2	0	1500	0	27	6.75	54	0.5	0.5
PCO-4	236.2	0	0	1500	27	6.75	54	0.5	0.2
PCO-5	236.2	0	1050	450	27	6.75	54	0.5	0.4
PCO-6	236.2	0	450	1050	27	6.75	54	0.5	0.2

**Table 6:** The percentage of voids by size in the cross-section of pervious concrete.

**Size (mm)**	**Porosity (%)**						
	**PCO-C**	**PCO-1**	**PCO-2**	**PCO-3**	**PCO-4**	**PCO-5**	**PCO-6**
<2	3.2	3.4	2.2	2.7	2.5	2.6	2.2
2–8	4.4	4.3	3.9	4.7	4.6	4.0	5.9
>8	3.7	3.9	6.8	8.1	8.8	5.7	6.4
Total	11.3	11.6	12.9	15.5	15.9	12.3	14.5

**Size (mm)**

**Porosity (%)**

**PCO-C**

**PCO-1**

**PCO-2**

**PCO-3**

**PCO-4**

**PCO-5**

**PCO-6**

**Table 7:** EDS results of S1-S4 areas (%).

Specimen	O	Na	Al	Si	K	Ca	C
PCO-C before S_1_	49.4	0.18	0.05	0.72	0.17	49.16	0.32
PCO-1 before S_2_	45.9	0.13	0.23	0.88	0.09	51.78	0.99
PCO-C after S_3_	54.98	0.36	1.58	11.21	0.25	30.65	1.01
PCO-1 after S_4_	46.08	0.21	0.34	1.02	0.05	42.93	1.53

**Table 8:** Results of XRD tests.

Intensity	PCO-C			PCO-1		
	Portlandite	Alite	Belite	Portlandite	Alite	Belite
Before the test (a.u.)	115	111	94	105	95	81
After exposure to water stream (a.u.)	109	85	76	113	91	80

**Table 9:** Changes in water parameters.

Water Parameters	CaO	El.l., µS	pH
Before the test (mg/L)	1	434	7.8
After exposure to water flow PCO-C (mg/L)	11	972	8.4
After exposure to water flow PCO-1 (mg/L)	8	709	8.3

**Table 10:** Freeze–thaw resistance results for pervious concrete.

Indicator	PCO-C	PCO-1	PCO-4	PCO-6
Mass change (%)	0	+0.5	+1.5	+1.8
Decrease in compressive strength (%)	31	3.3	4.7	4.2

**Table 11:** Single value of sound absorption.

Composition	PCO-C	PCO-1	PCO-4	PCO-6
DL_α_ (dB)	2.10	2.30	2.16	2.04
